# Perceptions of the barriers, facilitators, outcomes, and helpfulness of strategies to implement screening, brief intervention, and referral to treatment in acute care

**DOI:** 10.1186/s13012-021-01116-0

**Published:** 2021-04-23

**Authors:** Alyson Keen, Kelli Thoele, Ukamaka Oruche, Robin Newhouse

**Affiliations:** 1 Indiana University School of Nursing, 600 Barnhill Dr., Indianapolis, IN 46202 USA; 2 Indiana University Health Adult Academic Health Center, 1701 N. Senate Ave, Indianapolis, IN 46202 USA

**Keywords:** Screening, Brief Intervention, Referral to Treatment (SBIRT); Addiction; Substance-related disorders, Implementation, Strategies

## Abstract

**Background:**

Screening, Brief Intervention, and Referral to Treatment (SBIRT) is a clinical intervention used to address alcohol and illicit drug use. SBIRT use has resulted in positive health and social outcomes; however, SBIRT implementation remains low. Research on implementing interventions, such as SBIRT, lacks information about challenges and successes related to implementation. The Expert Recommendations for Implementing Change (ERIC) provides a framework to guide comprehension, clarity, and relevance of strategies available for implementation research. This framework was applied to qualitative feedback gathered from site coordinators (SCs) leading SBIRT implementation. The purpose of this study was to describe the SCs’ experiences pertaining to SBIRT implementation across a health system.

**Methods:**

Within the context of a larger parent study, a semi-structured interview guide was used to capture 14 SCs’ perceptions of the barriers, facilitators, and outcomes pertaining to SBIRT implementation. Qualitative data were analyzed using standard content analytic procedures. A follow-up survey was developed based on 14 strategies identified from qualitative data and was administered electronically to determine the SC’s perceptions of the most helpful implementation strategies on a scale of 1 (least helpful) to 5 (most helpful).

**Results:**

All 14 invited SCs participated in the SBIRT implementation interview, and 11 of 14 (79%) responded to the follow-up survey. Within the categories of barriers, facilitators, and outcomes, 25 subthemes emerged. The most helpful implementation strategies were reexamining the implementation (*M* = 4.38; *n* = 8), providing ongoing consultation (*M* = 4.13; *n* = 8), auditing and providing feedback (*M* = 4.1; *n* = 10), developing education materials (*M* = 4.1; *n* = 10), identifying and preparing champions *(M* = 4; *n* = 7), and tailoring strategies *(M* = 4; *n* = 7).

**Conclusion:**

SCs who led implementation efforts within a large healthcare system identified several barriers and facilitators to the implementation of SBIRT. Additionally, they identified clinician-related outcomes associated with SBIRT implementation into practice as well as strategies that were helpful in the implementation process. This information can inform the implementation of SBIRT and other interventions in acute care settings.

Contributions to the literature

- This study examined site coordinators’ (SCs) perceptions of implementing a clinical intervention by applying the Expert Recommendations for Implementing Change (ERIC) framework to identify commonly used and helpful implementation strategies.
- Study findings highlight barriers (e.g., sustainment, negative attitudes), facilitators (e.g., leveraging interdisciplinary support, adapting intervention to organizational context), and outcomes (e.g., increased awareness, action-oriented approach) perceived by SCs while implementing SBIRT, such findings can be considered by other change leaders preparing for intervention implementation.
- Considering there are 73 strategies in the ERIC framework, identifying implementation strategies most frequently used and most helpful (e.g., purposely reexamine the implementation, provide ongoing consultation) will help both researchers and clinicians prioritize strategies for future implementation projects or research.
- While this study identified the perceived helpfulness of multiple implementation strategies, future studies are needed to understand the mechanisms of action for specific implementation strategies deemed helpful to change leaders.

## Background

The addiction epidemic has plagued the USA, with 20.3 million Americans affected and an annual economic burden of $740 billion [1, 2]. Health-related consequences of addiction range from appetite, sleep, and mood changes to heart attack, stroke, overdose, and death [3]. Approximately 70,237 overdose deaths resulted from addiction in the USA in 2017 [4]. Of the 21.2 million Americans needing treatment in 2018, only 11.1% received specialty addiction treatment [1].

Screening, Brief Intervention, and Referral to Treatment (SBIRT) is endorsed by the Substance Abuse and Mental Health Services Administration [5] as an intervention to address alcohol and illicit drug use [6]. SBIRT has been implemented in multiple healthcare settings [7], including acute care, where patients are typically seeking care for health issues unrelated to addiction. In particular, SBIRT implementation on medical surgical units is associated with a decrease in risky alcohol use [8, 9]. Although SBIRT is associated with positive health and social outcomes [10–12] as well as positive clinician outcomes (e.g., increased SBIRT skills, improved self-efficacy) [5, 13], SBIRT implementation remains low [11, 12].

Despite a growing understanding of how and why interventions are adopted, implemented, and sustained, the concepts and descriptions of implementation strategies have been inconsistent in the literature. The Expert Recommendations for Implementing Change (ERIC) definitions by Powell et al. provides a framework to organize implementation strategies and inform comprehension, clarity, and relevance of 73 strategies to be considered as options for use for implementation research [14]. Commonly referenced strategies include tailored implementation strategies, educational outreach, printed education materials, local champion leaders, educational meetings, audit and feedback, and computerized reminders. However, more work is needed to clearly identify strategies and their associated mechanism of action that facilitate and enhance implementation from the lens of clinicians implementing change [15].

To begin to fill this gap, qualitative interview data were gathered from local champions (i.e., site coordinators) leading SBIRT implementation (i.e., parent study) in a large health care system in a Midwestern state in the USA. Responses were categorized into the implementation strategies described by Powell et al. [14]. Findings will enhance implementation scientists’ understanding of strategies commonly used and perceived by clinicians (nurses in this case) as most helpful for implementing SBIRT in acute care settings.

The parent study was completed using a phased cluster randomized design to evaluate nurse-led SBIRT [16]. In this parent study, participating hospitals selected a nurse site coordinator (SC) at each facility championing SBIRT implementation and complete required study training. The 8-h training included content and competency validation on SBIRT and implementation strategies, as well as a discussion of health system change [17]. SCs at each hospital tailored the SBIRT intervention to their clinical site by addressing barriers identified in their baseline and implementation assessment and adapting strategies based on the unit context [18]. During monthly study team calls, various challenges and successes emerged from observations and feedback from the SCs. The purpose of this study was to describe the SCs’ experiences implementing SBIRT across the healthcare system. Specific aims were to describe SCs’ perceptions of (1) barriers, facilitators, and outcomes related to SBIRT implementation; and (2) strategies perceived as most helpful to implement SBIRT.

## Methods

The parent study took place on 14 medical surgical units (i.e., one per hospital). Hospital settings included academic health centers, community hospitals, and critical access hospitals. Study participants were 14 study nurse SCs in various positions including direct care clinical nurses (*n* = 8), clinical educators (*n* = 2), clinical nurse specialists (*n* = 2), a nurse case manager (*n* = 1), and a house supervisor (*n* = 1). Within the context of the parent study, a semi-structured interview guide was used to capture the 14 SCs’ experiences pertaining to barriers, facilitators, and outcomes of SBIRT implementation. The SQUIRE 2.0 Revised Standards for Quality Improvement Reporting Excellence were used for reporting [19].

### Data collection

The interview guide was sent to all SCs for reflection prior to the interview and then data were collected via one-on-one telephone interviews with a research assistant or via e-mail. Responses to interview questions were recorded in a Word file on the semi-structured interview guide. Participants responded to the following interview questions: (1) what factors were the most helpful in the implementation of SBIRT?; (2) What barriers to implementation did you encounter?; (3) In what ways did study activities improve, help, or hinder care in your facility? Although interviews were not audio-recorded, notes were recorded in a Word file. The implementation strategies mentioned during the interviews were then categorized by the research assistant into the strategies described by Powell et al. [14], and a follow-up survey was created based on 14 commonly identified strategies described by SCs in the qualitative interviews. The survey was administered electronically and SCs responded by indicating the strategies that they used during the study and ranking the most helpful strategies on a scale from 1 (least helpful) to 5 (most helpful). The interviews and follow-up surveys were conducted during the 2 months (June and July 2019) following completion of the 12-month parent study.

### Data analysis

Interview responses were compiled into an Excel spreadsheet. Responses were categorized into themes and sub-themes using standard content analysis procedures by grouping similar responses [20]. The number of participant statements reflecting each theme/sub-theme was quantified by a research assistant (a PhD student). A second research assistant (also a PhD student) then independently reviewed the notes from the interviews and categorized the responses into themes and sub-themes. The two research assistants then reviewed the data together and verbally resolved all discrepancies. Further validation was completed with the SC group. SCs were provided the final list of themes and subthemes to evaluate content accuracy and potential missing content from the de-identified interview data. The response size and average rating for each implementation strategy were calculated. Implementation strategies were ordered from highest to lowest average rating to identify which strategies were considered most helpful to the SCs.

## Results

### Interviews

All 14 SCs provided qualitative data regarding SBIRT implementation. Although phone interviews were requested from all site coordinators, two site coordinators (14%) preferred to send responses to the interview questions via email, while the remaining twelve (86%) site coordinators completed the interviews via phone. Phone interviews ranged from 15 to 45 min. Three major themes were pre-determined based on the aims of the study (implementation barriers, facilitators, and outcomes), and within these themes, 25 subthemes emerged. A detailed list of subthemes with examples is provided in Table 1 and the most common subthemes with quotes from SCs are provided below.

**Table 1:** Site coordinator perception of SBIRT implementation

Theme	Sub-theme (# mentions)	Examples
**Barriers** identified related to SBIRT implementation were:	Sustainment (10)	Process complacency; difficult to coordinate with multiple clinicians
	Data collection (9)	Difficult to navigate health record; data absent from record
	Staff adoption (9)	Nurse adherence to SBIRT; negative attitudes
	Unit operational challenges (8)	Leader turnover; new nurses on the unit
	SC execution (7)	SC felt alone in implementation efforts; SC role unclear
	Study rollout (7)	Trial originally set up on different unit; site coordinator changed
	Training coordination (6)	Finding a training schedule that worked for staff
	Brief Intervention (BI) (5)	Lack of understanding of intent; discomfort with BI process
	Patient-specific (4)	Patients did not see relevance or did not want help
	Patient referral (2)	Insurance challenges; lack of available referral sites
	Effort duplication (2)	SBIRT activities/documentation duplicated (nursing and social work)
**Facilitators** identified related to SBIRT implementation were:	Leveraging support (9)	Involvement of interprofessional stakeholders; use of early adopters
	Adapting intervention (8)	Ability to tailor/create resources; dedicated location for resources
	SC development (8)	Learning and peer support from other SC; research team mentorship
	Feedback loop (8)	Auditing/follow-up; reminders; rounding with staff for questions
	Leader impact (7)	Leader engagement/support; SBIRT considered mandatory
	Sustainment (3)	Problems anticipated ahead of time; new hire assimilation
	Implementation efficiency (3)	Implementation planning phase; designated role (SC)
**Outcomes** identified related to SBIRT implementation were:	Awareness (13)	Clinician and patient awareness of risky substance use
	Action-oriented process (11)	Ability to act on positive screen; more referral options accessible
	Enhanced care transitions (8)	Streamline continuum of care; support collaborative communication
	Therapeutic relationships (6)	Show clinician care; determine patient readiness in the process
	Connection to disease process (6)	Applicability of BI to other conditions; prevention of alcohol withdrawal
	SC development (4)	Implementation skills; organizational resource navigation
	Comfort (4)	Clinician and patient comfort discussing substance use

#### Theme 1: implementation barriers

Participants described several factors that hindered the timely and seamless implementation of SBIRT. The most common barriers were challenges sustaining the implementation of SBIRT, collecting data from the electronic medical record to evaluate implementation, and staff nurse resistance to the use of SBIRT due to negative attitudes regarding the intervention. While reflecting on barriers to implementation, one participant stated, “Sustainment was difficult because the process was on paper and not yet integrated into the electronic medical record. Our providers and nurses are still asking about substance use but not with a validated assessment tool.” Another participant stated:

“Some nurses on the floor were kind of freaked out by a new process. Staff felt like they were getting thrown a curve (ball) adding onto the admission process and were feeling anxious. I had to keep reassuring staff that they were making it harder than it was.”

#### Theme 2: implementation facilitators

Participants also described several factors or resources that enhanced the timely and seamless implementation of SBIRT. The most common facilitators were leveraging support from staff nurses and interprofessional team members on the unit, adapting the intervention to fit the organizational context, and training and available support for SCs. One participant, reflecting on facilitators to implementation, stated, “Site coordinator training, supporting training resources, including power points, were super helpful. We also were able to modify (training materials) to meet our facility needs.” Another participant stated:

“Education and guidelines that were provided by the study team were helpful. The collaborative work that we did through the monthly calls helped me to make sure that I was on the right track by (making me) aware of things I needed to work on*.”*

#### Theme 3: implementation outcomes

Participants described the outcomes of SBIRT implementation for nurses. Benefits to nurses on the unit included increased awareness of substance use risks amongst their patients, the inclusion of an action-oriented approach for patients with risky substance use, and enhanced care transitions across clinicians and care settings. Nurses gained the confidence to engage actively with patients, leading to more trusting and therapeutic relationships. Ultimately, increased awareness and confidence helped nurses feel more prepared to intervene with patients with addiction. As one participant stated, “SBIRT raised education and awareness of the clinicians. We were able to provide better resources and identify patients needing support. Patients were falling through the cracks before.” Another participant said

“I think it gave us more of an awareness of patients’ backgrounds. Many times, we

do not get the whole history, and it was helpful to collect additional information about factors that could be contributing to poor health. The process gave us an opportunity to provide more resources based on data collected.”

### Survey

**Fig. 1:**
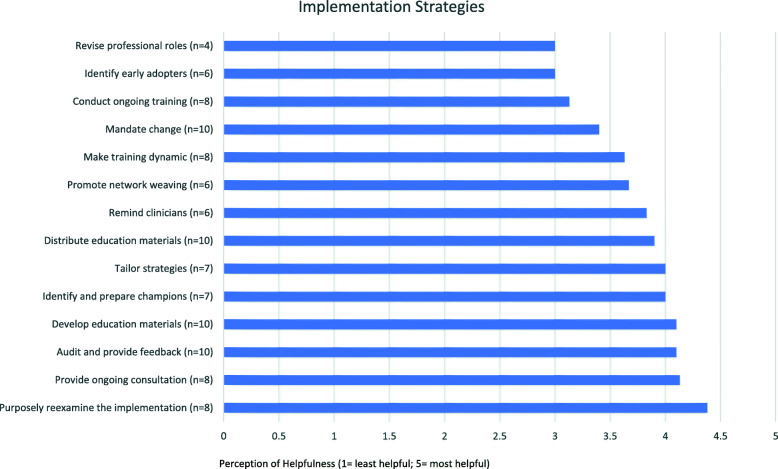
Site coordinator perceptions of commonly used implementation strategies

Eleven (79%) of the participants completed the follow-up survey. Figure 1 shows the most highly rated implementation strategies. According to participants, the most helpful (i.e., effective) implementation strategies were purposely reexamining the implementation (*M* = 4.38; *n* = 8), provide ongoing consultation (*M* = 4.13; *n* = 8), audit and provide feedback (*M* = 4.1; *n* = 10), develop education materials (*M* = 4.1; *n* = 10), identify and prepare champions (*M* = 4; *n* = 7), and tailor strategies (*M* = 4; *n* = 7).

## Discussion

Acute care clinicians need to be prepared to use clinical interventions like SBIRT considering the increase in substance use and abuse; however, use in acute care units is limited. Using the framework provided by Powell et al. [14], this study examined SCs’ perceptions of commonly used and helpful strategies for implementing SBIRT into acute care units. In the parent study, SCs were highly engaged in the SBIRT implementation and thus were able to provide insight into perceived challenges and successes of implementation. Based on the interviews, SCs identified barriers (e.g., challenges sustaining the implementation of SBIRT), facilitators (e.g., leveraging support from interprofessional team members), and outcomes (e.g., increased awareness of substance use risk) of SBIRT implementation. Among the implementation strategies, participants perceived that purposely reexamining the implementation, providing ongoing consultation, and auditing and providing feedback were the most helpful. Our study findings suggest that, although SCs experienced barriers to the implementation of SBIRT, the outcomes associated with SBIRT provided benefits to nurses and include increased awareness of and confidence in addressing substance use and potential to positively impact overall treatment for patients with substance use.

Several SCs were new to implementation. However, they expressed that many implementation strategies helped enhance the implementation process. Our findings are consistent with other SBIRT implementation research studies that used implementation strategies as defined by Powell et al. [14], including audit and provide feedback [8, 21, 22], develop education materials [21, 23], and tailor strategies [24]. Two strategies, audit and provide feedback, and tailor strategies, were rated among the most helpful and commonly used, similar to findings in a review of utilized implementation strategies [15]. All SCs voiced the value of available training resources, study team mentorship, and peer support as they moved through the implementation process. Given their experience, many SCs stated that they felt more confident leading change in the future due to the knowledge and skills they developed during SBIRT implementation.

SCs reported that it was important to leverage support from other interprofessional team members, such as social workers and clinical educators. Specifically, they noted the opportunity to identify additional stakeholders (e.g., unit secretary, peer coaches) and better align existing resources in preparation for SBIRT implementation. Many of the SCs had not implemented or used SBIRT, nor were they able to identify who delivered brief interventions or referral to treatment when needed. In follow up, site interviews were initiated to identify who delivered each phase of the clinical intervention [18]. Challenges with identifying stakeholders and aligning resources also might have been due to some of the SCs’ limited leadership experiences. SCs may not have been aware of all of the resources available at their facilities or had experience leading interdisciplinary work. In the future, training should include exercises that help SCs to think through the SBIRT process and identify stakeholders as a group. These exercises may facilitate a deeper understanding of the stakeholders involved in SBIRT implementation and more comprehensive stakeholder engagement.

SCs reported that the sustainability of the SBIRT intervention was one of the primary barriers. Sustainability has been described as the ongoing use and evolution of a practice change within an environment over time [25]. According to SCs, integrating a new change into practice was challenging. SCs sensed process complacency as the study started to wind down, which some described as common with newly implemented change. As the study ended, the next steps for SBIRT integration and spread throughout the system were unclear. With the absence of structure previously provided by the study team, SBIRT practice was difficult to sustain. For example, monthly meetings with the study team and routine study-related data collection activities motivated SCs to continue monitoring SBIRT implementation. Planning and measurement of sustainability are complex, with many not understanding the difference and the overlap between implementation and sustainability [25]. The lessons learned from implementing SBIRT included the importance of adapting clinical interventions to the environment and identifying expert stakeholders to partner with during the implementation process. However, an opportunity exists for strategic sustainability planning in early study phases to ensure that the clinical practice continues. While this study identified the perceived helpfulness of multiple implementation strategies, a systematic investigation is needed to understand why specific implementation strategies are helpful to change leaders.

## Limitations

Limitations of this study include the small sample size, participant bias, and the interview approach. This study involved interviews from a small sample of SCs; albeit this is typical of quality improvement feedback when implementing changes within health systems. The respondents were all SCs and received similar training, which potentially biased responses to similar implementation processes. Interviewing additional staff members responsible for intervention delivery may have led to the identification of other themes or provided a more comprehensive identification of experiences and feedback related to the SBIRT implementation. Interviews were conducted over the telephone or e-mail and were not audio-recorded. Consequently, we were not able to capture some nonverbal data that may have contributed to the analysis, and we were limited to field notes collected during the interviews. Additionally, the semi-structured approach used in the interviews may have limited generation of additional data related to SBIRT implementation.

## Conclusion

SCs who led implementation efforts within a large healthcare system identified several barriers and facilitators to the implementation of SBIRT. Additionally, they identified nurse-related outcomes associated with the implementation of SBIRT into practice and strategies that were helpful in the implementation process. This information can inform the implementation of SBIRT and other interventions in acute care settings.

## Data Availability

The data generated and analyzed during the study are available from the corresponding author upon reasonable request.

## Abbreviations

ERIC: Expert Recommendations for Implementing Change
SBIRT: Screening, Brief Intervention, and Referral to Treatment
SC: Site coordinator
